# Arteriovenous Fistula Maturation Failure in a Large Cohort of Hemodialysis Patients in the Netherlands

**DOI:** 10.1007/s00268-017-4382-z

**Published:** 2017-11-29

**Authors:** Bram M. Voorzaat, Koen E. A. van der Bogt, Cynthia J. Janmaat, Jan van Schaik, Friedo W. Dekker, Joris I. Rotmans, on behalf of the Dutch Vascular Access Study Group, Bram M. Voorzaat, Koen E. A. van der Bogt, Cynthia J. Janmaat, Jan van Schaik, Friedo W. Dekker, Joris I. Rotmans, Liffert Vogt, Laurens Huisman, Bas A Th. F. Gabreëls, Frans T. J. Boereboom, Irene M. van der Meer, Randolph G. S. van Eps, Daniël Eefting, Marcel C. Weijmer, Roos C. van Nieuwenhuizen, Alferso Abrahams, Raechel J. Toorop

**Affiliations:** 1 Department of Internal Medicine, Leiden University Medical Center, Albinusdreef 2, 2333 ZA Leiden, The Netherlands; 2 Department of Surgery, Leiden University Medical Center, Leiden, The Netherlands; 3 Department of Surgery, Haaglanden Medical Center, The Hague, The Netherlands; 4 Department of Clinical Epidemiology, Leiden University Medical Center, Leiden, The Netherlands

## Abstract

**Objectives:**

Radiocephalic arteriovenous fistulas (RCAVF) are the preferred vascular access (VA) for hemodialysis (HD). Cohort studies from North America revealed that nonmaturation is a significant disadvantage of RCAVFs compared to other VAs. DESIGN: This present retrospective study describes the incidence of nonmaturation of AVFs and functional failure of arteriovenous grafts (AVG) in a multicentre cohort in the Netherlands and attempts to create a prediction model for nonmaturation of RCAVFs. Furthermore, the efficacy of interventions to promote maturation as well as the variability between hemodialysis centers was evaluated.

**Materials:**

Medical records from 8 hospitals from 1997 to 2016 were retrospectively evaluated for VA type, maturation/primary success and demographics and comorbidities.

**Methods:**

A prediction model was created for RCAVF nonmaturation using multivariate logistic regression analysis, selecting significant predictors using backward selection. Discrimination and calibration of the model were assessed.

**Results:**

1383 AVFs and 273 AVGs were included in 1221 patients. Overall nonmaturation was 24% for RCAVFs, and 11% for upper arm AVFs. The functional failure rate for AVGs was 6%. The nonmaturation rate of contralateral RCAVFs after failure of an RCAVF was 22%. Procedures to improve RCAVF maturation were successful in 98/142 cases (69%). Predictors for nonmaturation were female gender, peripheral vascular disease, cerebrovascular disease and a cephalic vein diameter <2.5 mm, but the prediction model lacked sensitivity and specificity predicting individual RCAVF nonmaturation (C-statistic 0.629).

**Conclusion:**

Nonmaturation rates are highest for RCAVFs, but nonmaturation could not be predicted with demographic parameters.

**Electronic supplementary material:**

The online version of this article (10.1007/s00268-017-4382-z) contains supplementary material, which is available to authorized users.

## Introduction

The arteriovenous fistula (AVF) is the preferred type of permanent vascular access (VA) in maintenance hemodialysis (HD) patients. AVFs are associated with a lower incidence of patency-related procedures than arteriovenous grafts (AVGs) and less infectious complications than both AVGs and central venous catheters (CVC). As a consequence, healthcare costs are lowest for patients with an AVF, compared to patients with an AVG or CVC [1].

Both the NKF KDOQI and EBPG guidelines advocate the creation of AVFs distally in the upper extremity whenever possible [1, 2]. Radiocephalic AVFs (RCAVFs) have the advantage of preservation of more proximal options for future VAs in case of access failure. In addition, RCAVFs are associated with a lower incidence of HD access-induced distal ischemia [3], when compared to upper arm AVFs. High flow also predisposes to increased cardiac output and impaired systemic blood flow in patients with impaired cardiac function, a phenomenon known as ‘AVF cardiotoxicity’ [4, 5].

The main disadvantage of RCAVFs is nonmaturation, characterized by inadequate dimensions of the venous outflow tract or insufficient blood flow [6]. Although a uniform definition of nonmaturation is lacking, rates up to 65% are reported [7]. Forearm location and female gender are well-known risk factors for early failure [8]. A decade ago, Lok and co-workers [9] developed a scoring system to predict nonmaturation in a North American cohort. Predictors were age over 65 years, female gender, non-white race, and coronary and peripheral arterial disease.

Most studies on AVF maturation are from the USA and Canada. As demonstrated in the DOPPS study, CVC preference is higher [10] and AVF cannulation is performed later [11] than in Europe. Other significant differences are ethnicity, BMI and cardiovascular comorbidities [12]. The aim of the current study was to evaluate the incidence of nonmaturation of RCAVFs and upper arm AVFs in a large cohort in the Netherlands and to create a prediction model for RCAVF nonmaturation. As a comparator group, functional failure of AVGs was also assessed. In addition, the efficacy of interventions to promote maturation as well as the variability between HD centers was assessed.

## Methods

### Patient selection

Adult patients who underwent creation of an AVF or AVG as a permanent VA for maintenance HD were retrospectively identified in 5 affiliated teaching hospitals and 3 academic hospitals in the Netherlands. To prevent survivorship bias, the time frame varied per hospital and was limited to years in which medical records were available for all consecutive AVF and AVG recipients in that year (Supplemental Table 1). Overall, patients receiving their VA between 1997 and 2016 were included.

The Medical Research Involving Human Subjects Act (WMO) was not applicable. Ethical approval was granted by the medical ethics committees of the Leiden University Medical Center. Data were collected and processed in accordance with the local research code of conduct.

### Data collection

Data were collected from clinical records and included demographic variables, comorbidities, medication use, laboratory results, VA configuration and surgical details, initiation and abandonment of VA use, ultrasound results, surgical and endovascular interventions and clinical adverse events. Ethnicity of patients was not registered due to objections by the ethical committee.

### Outcomes and candidate predictors

Preemptively created VAs were defined as VAs created in a patient who did not receive HD within 2 weeks after VA creation. The VA was considered mature if it was successfully used for at least three consecutive HD sessions or if the Robbin’s ultrasound criteria for maturation were met [13]. The VA was considered nonmature if it was not cannulated in a patient on HD. If the patient has not started HD, a VA was considered nonmature if ultrasound or angiography demonstrated a failed VA using Robbin’s criteria or another VA was created. If maturation could not be assessed due to death, kidney transplantation or loss to follow-up before VA cannulation or ultrasound, it was considered indeterminate.

For prevalent HD patients, maturation time was defined as the time until cannulation or ultrasound demonstrating maturation, whichever came first. Assisted maturation was defined as maturation with a procedure to improve patency.

A list of candidate predictors for nonmaturation was compiled: patient age over 60 years, female gender, diabetes mellitus, a body mass index (BMI) over 25 kg/m^2^, symptomatic coronary, cerebrovascular or peripheral arterial disease, an ipsilateral central venous catheter, hypertension, cystic kidney disease, whether the fistula was created preemptively and a preoperative diameter of the artery or vein below 2.5 mm.

### Statistical analysis

Statistical analyses were performed for RCAVFs, upper arm AVFs and upper extremity AVGs. *T*- and χ^2^-tests were used where applicable. Baseline characteristics were summarized as mean with standard deviations for continuous variables and as count with percentages for categorical variables. Missing data were handled by multiple imputation methods using fully conditional specification with 10 repetitions [14, 15]. Candidate predictors, VA sidedness and maturation outcome were entered. For age, BMI, mean arterial pressure and artery and vein diameters, continuous values were entered into the multiple imputation. The imputed values were dichotomized to appropriate categories.

A prediction model for nonmaturation was created. Candidate predictors were entered in a multivariate logistic regression analysis, with nonmaturation as the dependent variable. Backward selection was used to identify the most significant independent predictors. In logistic regression analysis, candidate predictors were considered significant at a *p* value <0.30. *p* value of 0.30 was applied as conservative selection criterion to limit chances of overfitting [16]. We used the majority method to select the predictors for the final prediction model [17]. Predictors significant in at least 7 out of 10 imputation sets were entered into the final logistic regression analysis. Subsequently, forward selection was used to check stability of the results.

Sensitivity analysis was performed by repeating the logistic regression analysis with a significance level of *p* value <0.40, <0.25 and <0.20. The model’s predictive performance was examined by estimating calibration and discrimination. A receiver operating characteristic analysis was performed for the model, and C-statistics from all imputation sets were pooled [18]. Statistical analysis was performed using IBM SPSS Statistics version 22 (IBM Corp., Armonk, NY).

## Results

### Patient characteristics and VA configurations

**Fig. 1:**
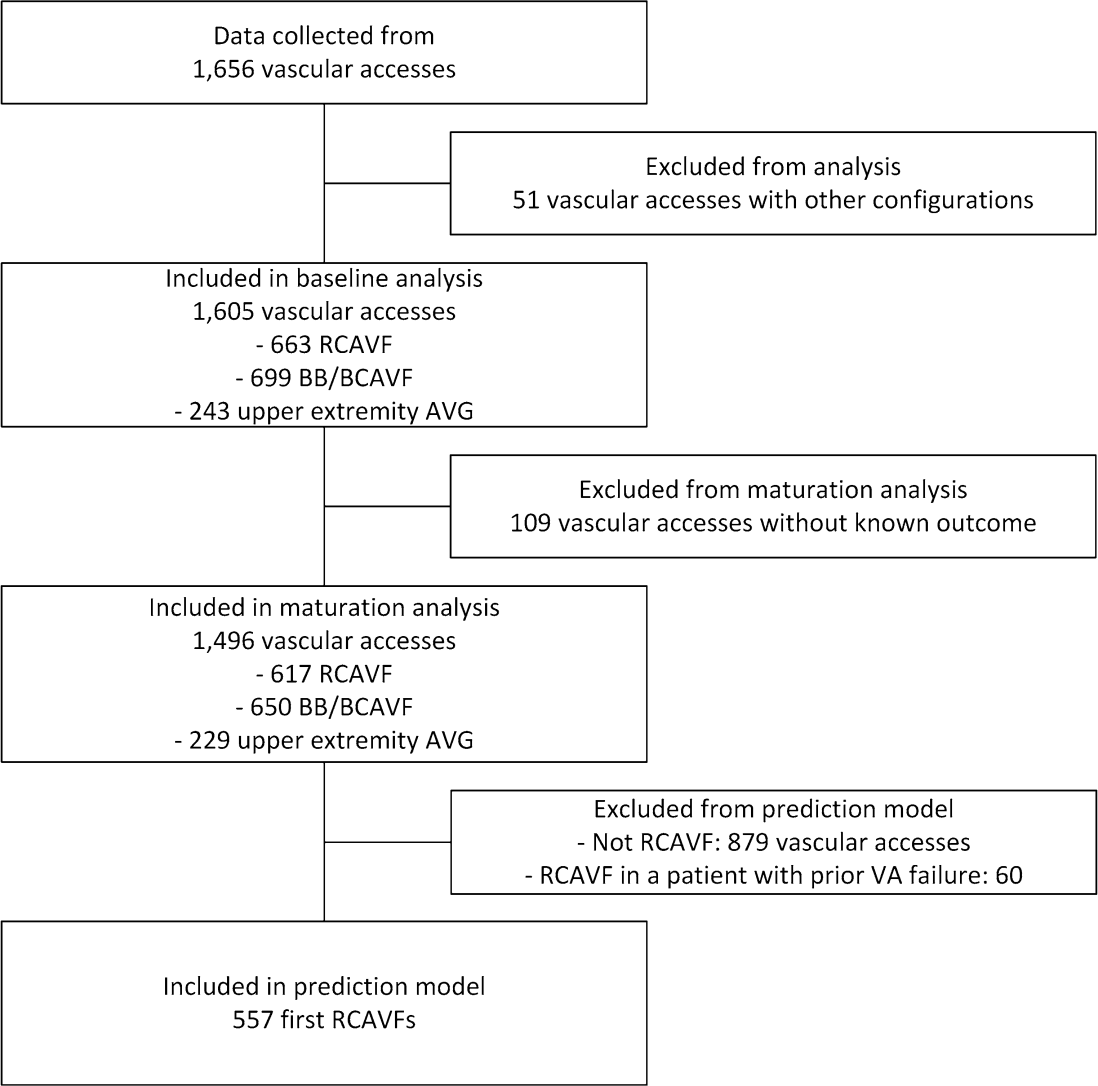
Flow chart demonstrating exclusion of VAs from analysis

Data from 1656 VAs (1383 AVF and 273 AVG) in 1 221 patients were obtained (Table 1). RCAVFs and upper arm AVFs and AVGs were the most common configurations. The 51 other configurations constituted 3.1% of the cohort and were excluded from the analysis (Fig. 1). The earliest VA available in the cohort was created in 1997 (Supplemental Table 1). Baseline measurements for arterial and venous diameters were missing in 43 and 25%, respectively, in cases where diameters were only described as ‘suitable’ in clinical practice. Additionally, the perioperative mean arterial pressure was unknown for 12.1% of cases and the BMI was missing for 7.5%

**Table 1:** Patient characteristics at vascular access creation

	RCAVF	Upper arm AVF	AVG	*p* value		
				RCAVF versus other	Upper arm AVF versus other	AVG versus other
Number of accesses *n* = 1605	663	699	243			
Gender						
Male	463 (69.8%)	363 (51.9%)	108 (44.4%)	<0.001	<0.001	<0.001
Female	200 (30.2%)	336 (48.1%)	135 (55.6%)			
Patient age	62.6 ± 15.3 year	62.7 ± 14.6 year	64.3 ± 14.9 year	0.516	0.624	0.116
Body mass index (BMI)	27.1 ± 5.9 kg/m^2^	26.5 ± 6.2 kg/m^2^	27.6 ± 6.5 kg/m^2^	0.288	0.017	0.066
Mean arterial pressure at VA creation	103 ± 16.4 mmHg	96 ± 17.3 mmHg	96 ± 18.4 mmHg	<0.001	<0.001	0.003
First permanent vascular access for patient	596 (89.9%)	414 (59.2%)	113 (46.5%)	<0.001	<0.001	<0.001
Preemptively created	366 (55.2%)	274 (39.2%)	83 (34.2%)	<0.001	<0.001	<0.001
Jugular catheter present				<0.001	<0.001	0.263
No catheter	381 (57.5%)	301 (43.1%)	108 (44.4%)			
Ipsilateral catheter	100 (15.1%)	145 (20.7%)	50 (20.6%)			
Contralateral catheter	182 (27.5%)	253 (36.2%)	85 (35.0%)			
Preoperative ultrasound						
Target vein diameter	2.9 ± 0.8 mm	3.9 ± 1.3 mm	3.9 ± 1.4 mm	<0.001	<0.001	<0.001
Artery diameter	2.6 ± 0.6 mm	4.3 ± 1.1 mm	4.5 ± 1.0 mm	<0.001	<0.001	<0.001
Cause of renal failure				0.163	0.092	0.112
Diabetes mellitus	132 (19.9%)	165 (23.6%)	72 (29.6%)			
Glomerulonephritis	72 (10.9%)	80 (11.4%)	22 (9.1%)			
Vascular disease	147 (22.2%)	140 (20.0%)	52 (21.4%)			
Interstitial nephropathy	43 (6.5%)	48 (6.9%)	14 (5.8%)			
Cystic kidney disease	50 (7.5%)	34 (4.9%)	14 (5.8%)			
Congenital/hereditary disease	17 (2.6%)	21 (3.0%)	7 (2.9%)			
Multisystem disease	29 (4.4%)	39 (5.6%)	5 (2.1%)			
Other	91 (13.7%)	109 (15.6%)	27 (11.1%)			
Unknown	82 (12.4%)	63 (9.0%)	30 (12.3%)			
Comorbid conditions						
Diabetes mellitus	242 (36.5%)	282 (40.3%)	128 (52.7%)	0.005	0.841	<0.001
Coronary artery disease	173 (26.1%)	182 (26.0%)	59 (24.3%)	0.818	0.845	0.558
Peripheral vascular disease	125 (18.9%)	127 (18.2%)	41 (16.9%)	0.603	0.937	0.545
Cerebrovascular disease	94 (14.2%)	97 (13.9%)	39 (16.0%)	0.844	0.649	0.406
Medication use						
Antiplatelet	227 (34.2%)	238 (34.0%)	77 (31.7%)	0.739	0.835	0.456
Anticoagulant	100 (15.1%)	139 (19.9%)	47 (19.3%)	0.016	0.057	0.501
ACE inhibitor	244 (36.8%)	240 (34.3%)	83 (34.2%)	0.300	0.465	0.679
Angiotensin receptor blocker	197 (29.7%)	188 (26.9%)	62 (25.5%)	0.162	0.453	0.378
Calcium channel blocker	292 (44.0%)	340 (48.6%)	113 (46.5%)	0.109	0.117	0.977
Aldosterone antagonist	15 (2.3%)	9 (1.3%)	4 (1.6%)	0.184	0.219	0.899
Phosphate binder non-calcium based	346 (52.2%)	400 (57.2%)	151 (62.1%)	0.012	0.343	0.033
Phosphate binder calcium based	282 (42.5%)	259 (37.1%)	105 (43.2%)	0.117	0.022	0.307
Vitamin D	519 (78.3%)	558 (79.8%)	187 (77.0%)	0.697	0.355	0.457
Corticosteroids	59 (8.9%)	97 (13.9%)	23 (9.5%)	0.016	0.002	0.364
Other immunosuppressant	19 (2.9%)	51 (7.3%)	9 (3.7%)	0.001	<0.001	0.341
Laboratory measurements						
Calcium	2.28 ± 0.18 mmol/l	2.27 ± 0.19 mmol/l	2.25 ± 0.20 mmol/l	0.191	0.670	0.234
Phosphate	1.67 ± 0.51 mmol/l	1.67 ± 0.54 mmol/l	1.65 ± 0.48 mmol/l	0.751	0.983	0.681
PTH	37.2 ± 46.6 pmol/l	35.7 ± 38.3 mmol/l	31.5 ± 25.3 mmol/l	0.273	0.999	0.138
HbA1c-IFCC	44.5 ± 13.5 mmol/mol	45.1 ± 13.2 mmol/mol	44.8 ± 16.3 mmol/mol	0.635	0.615	0.960

Females and patients with diabetes more frequently received an AVG and females more frequently received an upper arm AVF. Fifty-five percent of RCAVFs were preemptively created, compared to 39 and 34% for upper arm AVFs and AVGs, respectively. RCAVFs were most often the first VA, with 90% created in patients without a prior VA (Table 2).

**Table 2:** Timing of VA surgery for VA configurations

VA configuration (*n*) *n* = 1605	On HD at time of VA creation			First access for patient
	Yes	No but started within 3 months	No started after 3 months or never	
RCAVF (663)	44.8% (297)	16.6% (110)	38.6% (256)	89.9% (596)
BCAVF (547)	56.5% (309)	17.4% (95)	26.1% (143)	62.9% (344)
BBAVF (152)	76.3% (116)	8.6% (13)	15.1% (23)	46.1% (70)
AVG (243)	65.8% (160)	18.1% (44)	16.0% (39)	46.5% (113)

Postoperative ultrasound examinations were not routinely performed during the historical timeframe of the study and were available for 28% (448/1605) of VAs. For 1496 out of 1605 VAs (93.2%), the maturation outcome could be determined (Fig. 1 and Supplemental Table 2).

### Incidence of nonmaturation

The incidence of nonmaturation was 24% for RCAVFs. This was lower than the nonmaturation incidence of upper arm AVFs and functional failure of AVGs (*p* <0.001 for RCAVF versus upper arm AVF, Table 3). The short-term follow-up of VAs, defined as achieving 3 months or 6 months of functional patency, was similar for upper arm AVFs (3 months: 77.8%, 6 months: 69.5%) and AVGs (3 months: 77.7%, 6 months: 68.6%) and worse for RCAVFs (3 months: 66.6%, 6 months: 59.5%) (Supplemental Table 3).

**Table 3:** 6-week and 3-month cannulation rates and primary failure per VA configuration. Patients who did not initiate HD or did not use their VA for reasons unrelated to nonmaturation were excluded

	Patients on HD at time of VA creation			Started HD within 3 months	All VAs with known outcome *n* = 1496
	Use at 6 weeks	Use at 3 months	Time until use (days ± SD)	Use at 3 months	AVF nonmaturation/AVG functional failure
RCAVF	17.4% (50/287)	61.3% (176/287)	68 ± 44	81.1% (86/106)	24.1% (149/617)
Upper arm AVF	22.0% (89/404)	72.5% (293/404)	66 ± 43	93.5% (100/107)	10.6% (69/650)
AVG	71.0% (110/155)	91.6% (142/155)	31 ± 19	97.6% (41/42)	5.7% (13/229)

Unassisted maturation was lowest for RCAVFs, at 60% (370/617), versus 79% for upper arm AVFs. Assisted maturation could be achieved even after multiple procedures (Supplemental Table 4 and Supplemental Figure 1). Eighty percent of AVGs did not require procedures before first use.

**Fig. 2:**
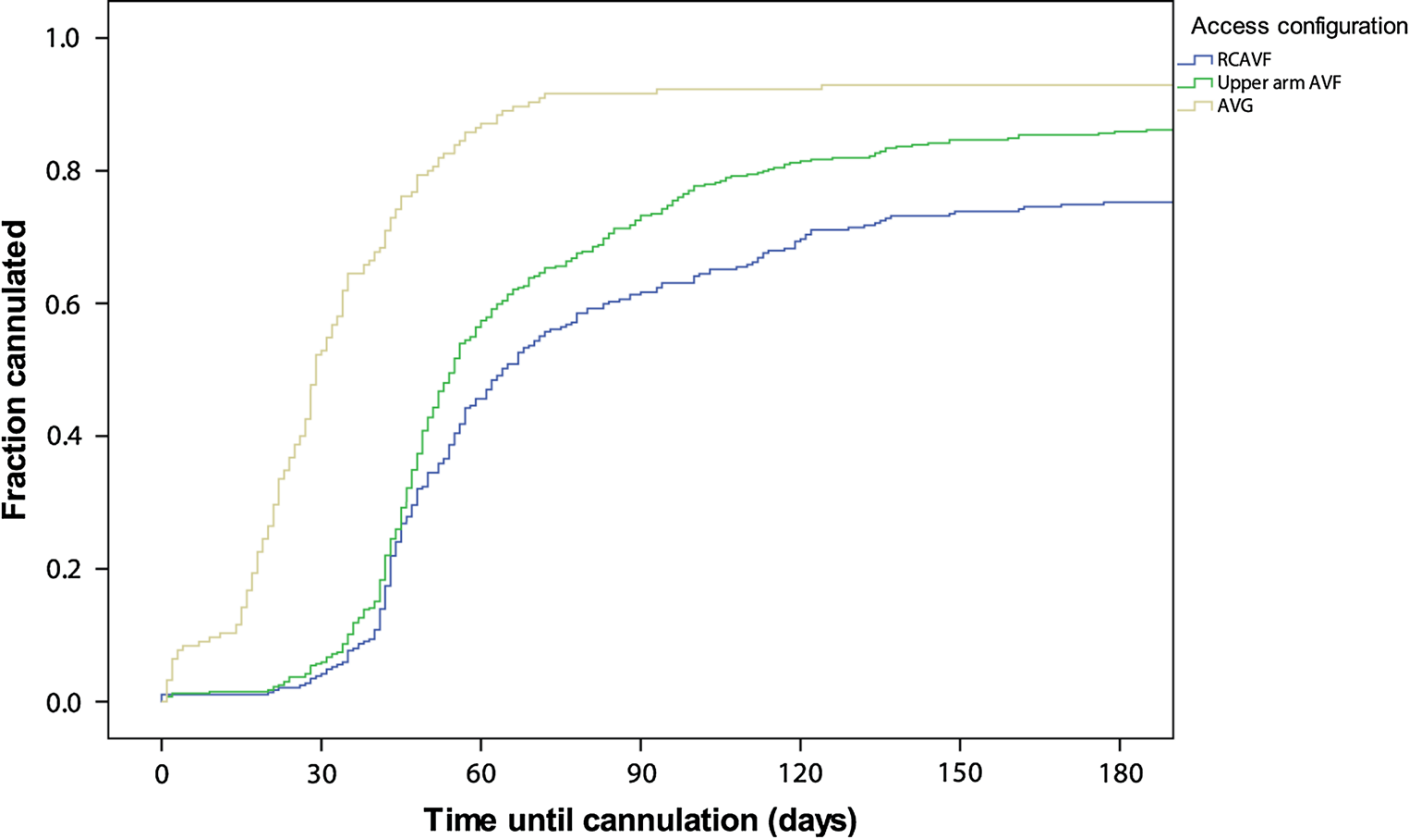
Time until first cannulation in patients prevalent on HD at the time of VA creation

Of RCAVFs preemptively created in patients who initiated HD within 3 months, 81% were cannulated within 3 months (Table 3). In prevalent HD patients, 61% of RCAVFs were cannulated within 3 months. AVGs were cannulated earlier than RCAVFs and upper arm AVFs, which were rarely used within 6 weeks (Table 3, Fig. 2). The 3-month cannulation rates in prevalent HD patients differed substantially between hospitals, ranging from 48 to 70% for RCAVFs and 33–80% for upper arm AVFs (Supplemental Table 5).

Over the timeframe of the study, no significant change in maturation of AVFs or primary success of AVGs was observed (Supplemental Table 6).

Fifty-nine patients received subsequent RCAVFs in both arms. Of the first RCAVFs, 34 (57%) did not mature, the remainder failed after initial successful use. Forty-one out of 59 (69%) subsequently created contralateral RCAVFs matured without procedures. As 5 RCAVFs reached maturation with procedures, the assisted maturation of these contralateral RCAVFs was 78%. Thirteen out of 59 (22%) RCAVFs failed due to nonmaturation. For 462 RCAVFs, the preoperative venous diameter and the maturation outcome were recorded (Table 1). Of RCAVFs with a recorded preoperative venous diameter of 2.5 mm or more, 225/295 (76%) were successful. From the group of AVFs with a preoperative venous diameter below 2.5 mm, 113/167 (68%) matured successfully (*p* = 0.045).

### Prediction of nonmaturation

**Fig. 3:**
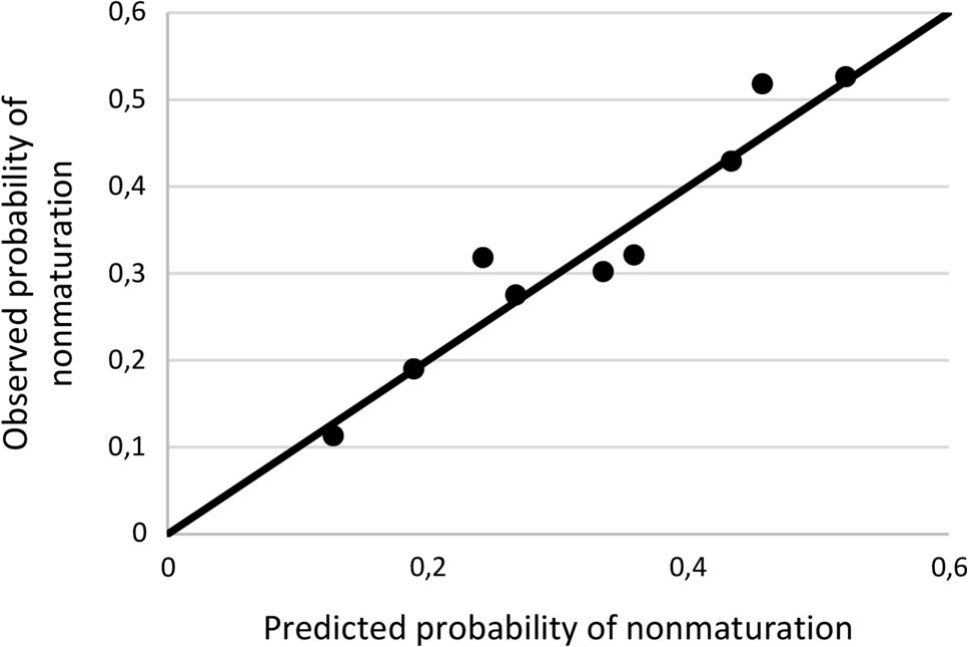
Calibration of the prediction model for nonmaturation of first RCAVFs

In the logistic regression analysis, 4 out of 13 predictor variables were significant at *p* <0.30 with backward selection in at least 7 of 10 imputed datasets (Table 4). In the sensitivity analysis restriction of the removal criterion for backward selection to *p* <0.25 removed the predictor peripheral vascular disease, while *p* <0.40 added the predictor arterial diameter <2.5 mm. These results were stable with forward selection. The risk equation of this model predicted RCAVF nonmaturation with a median area under the ROC-curve of 0.629 (interquartile range 0.626–0.633). Calibration of the model was assessed by comparing observed and predicted risk (Fig. 3).

**Table 4:** Predictors based on multivariate logistic regression analysis

Variable	Beta	Odds ratio (95% confidence interval)	*p*
Preoperative cephalic vein diameter <2.5 mm	0.426	1.53 (1.01–2.32)	0.044
Female gender	0.787	2.20 (1.47–3.29)	<0.001
Peripheral vascular disease	0.326	1.39 (0.84–2.28)	0.198
Cerebrovascular disease	−0.784	0.46 (0.23–0.89)	0.022

The intercept of the model was −1.452

## Discussion

In the present study, we retrospectively evaluated primary outcomes of 1656 VAs in a multicentre cohort of 1221 HD patients in the Netherlands. Comorbidities are comparable to previous American cohorts, whereas the BMI of patients in our cohort (27 kg/m^2^) is slightly lower, when compared to previous studies (28–30 kg/m^2^). [7, 12]. The proportion of preemptively created RCAVFs (55%) was higher than in Northern American studies ranging between 46 and 49% [7, 9].

### Incidence of nonmaturation

The 24% rate of primary failure of RCAVFs appears lower than the rates reported by Dember et al. (65%), Huijbrechts et al. (40%) and Schinstock et al. (37%) [7, 19, 20]. In the study by Dember et al. [7], 14% of AVFs were considered nonmature as determined by ultrasound criteria, although they were being used for HD. We found no improvement of AVF maturation over time.

It is important to notice that the definition of nonmaturation in our retrospective study differs from prospective studies. As follow-up ultrasound examinations were not routinely performed and a large proportion of AVFs was created preemptively, a composite measure of functional use and ultrasound criteria was created.

Although AVGs have a lower 5.7% incidence of functional failure than the nonmaturation incidence of upper arm AVFs (10.6%), this advantage is offset by the higher loss of AVG patency after cannulation, resulting in similar rates of 3- and 6-month functional patency.

### RCAVF versus other configurations

Like previous studies, we demonstrate that RCAVFs have the highest rate of delayed cannulation and nonmaturation. Over the duration of the study since 1997, no improvement of maturation has been observed. Our findings confirm the findings by Masengu, et al. [21] that age, gender and vascular disease are associated with, but do not reliably predict nonmaturation. In contrast, Lok, et al. [9] were able to predict nonmaturation in their model. Possible explanations are the different population in the USA and Canada and differences in patient selection and surgical practice, compared to Europe. Comparable to previous studies, we found a high rate of nonmaturation in females [22–24].

RCAVFs were commonly created in patients without a history of a failed VA. It is assumed that patients receiving an upper arm AVF as their first VA often had forearm vasculature not suitable for an RCAVF. It remains unclear whether this reflects local anatomical variations or a more generalized unsuitability of the patients’ vasculature. Based on our results, we hypothesize RCAVF nonmaturation is not solely explained by demographics and comorbidities. The anatomy of the RCAVF itself appears prone to nonmaturation.

If nonmaturation was strongly associated with comorbidities and demographics, one would expect a high nonmaturation rate of contralateral AVFs in individual patients with prior VA failure. In this respect, an important observation was the 22% primary failure rate of RCAVFs in patients with a non-matured contralateral RCAVF. Rather than being increased, at 22% this was similar to the overall 24% risk of RCAVF nonmaturation in our cohort. This illustrates that comorbidities do not explain nonmaturation substantially. One possible explanation is preferential creation of the first VA in the non-dominant arm, even if the vasculature of the dominant arm is more suitable (i.e., larger vessels).

### Interventions to promote maturation

Out of a total of 142 RCAVFs undergoing procedures to improve maturation, 98 (69%) matured. Although it cannot be ruled out that these also would have matured spontaneously, procedures to assist maturation appeared to be a worthwhile strategy to promote AVF usability. Similar results were observed by Shin et al. [25] achieving successful cannulation in 14 out of 19 cases (74%) of balloon angioplasty for AVF nonmaturation due to localized stenosis. In a study by Miller et al. [26] extensive balloon angioplasty and side branch interruption of 75 nonmature AVFs with a diameter of 2.0–5.0 mm resulted in successful cannulation of 71 AVFs after a median of 2.6 procedures.

### Variability among hospitals

In our cohort, patients from both academic and referral hospitals were included. The variability in maturation rates of AVFs among centers was remarkable. Based on the current data, it cannot be determined whether these differences result from the process of care or demographic characteristics of the patients that we did not include in our analysis.

### Limitations

Due to the retrospective design, the maturation outcome could not be determined for 10% of VAs. Another limitation of the current study is the unavailability of routine 6-week ultrasound examinations. Postoperative ultrasound examinations were often performed for symptoms or suspected nonmaturation. These therefore cannot be extrapolated to the entire cohort.

The time until first cannulation in prevalent hemodialysis patients should be interpreted with caution. As Robbin, et al. [27] demonstrated, most of the maturation occurs within 2 weeks after surgery. We cannot distinguish if the differences between the 6-week and 3-month cannulation rates of 17 and 61%, respectively, reflect actual delayed maturation or clinicians’ reluctance to early cannulation. Only a prospective study in which serial ultrasound examinations or early cannulation attempts are performed can reliably assess the potential for early cannulation of AVFs.

As the weak prediction model did not result in a clinically applicable risk equation, we did not perform external validation. One limitation could be the lack of data on ethnicity, an important factor in the scoring system by Lok et al. [9].

### Future directions

One approach to prevent nonmaturation is careful patient selection. New strategies are needed to identify patients at high risk of nonmaturation. A shift toward upper arm AVFs as the primary VA option seems attractive. However, losing distal VA options may not be acceptable for all patients and high-flow symptoms more often occur with upper arm AVFs. Therefore, such paradigm shift seems not to be the right solution.

## Conclusion

While the AVF has the best long-term outcome, the choice of VA should be tailored for each individual patient. Clinicians should weigh the benefits of future options and a lower incidence of high-output symptoms in RCAVFs to the risk of nonmaturation. This study demonstrates that for patients clinically eligible to receive an RCAVF, demographic parameters and comorbid conditions explain only a small part of AVF nonmaturation. In case of a failed RCAVF, a new RCAVF at the contralateral arm should not be avoided if the vasculature is suitable.

## Electronic supplementary material

Below is the link to the electronic supplementary material.

Supplementary material 1 (TIFF 1280 kb)

Supplementary material 2 (DOCX 14 kb)

Supplementary material 3 (DOCX 16 kb)

Supplementary material 4 (DOCX 16 kb)

Supplementary material 5 (DOCX 16 kb)

Supplementary material 6 (DOCX 16 kb)

Supplementary material 7 (DOCX 17 kb)
