# Supplementary material for: Arteriovenous Fistula Maturation Failure in a Large Cohort of Hemodialysis Patients in the Netherlands
Source: World J Surg. 2017 Nov 29;42(6):1895–903. doi: 10.1007/s00268-017-4382-z (PMC5934452; doi:10.1007/s00268-017-4382-z)
Supplement: Supplementary file 2 — Supplementary material 2 (DOCX 14 kb) [file 268_2017_4382_MOESM2_ESM.docx]

| **Hospital** | **RCAVF (n)** | **Upper arm AVF (n)** | **AVG (n)** | **Time frame** |
| --- | --- | --- | --- | --- |
| **1** | 69 (53%) | 55 (42%) | 6 (5%) | 2004-2015 |
| **2** | 99 (39%) | 56 (22%) | 101 (40%) | 2002-2015 |
| **3** | 47 (23%) | 144 (69%) | 17 (8%) | 2008-2015 |
| **4** | 60 (48%) | 53 (43%) | 11 (9%) | 1997-2015 |
| **5** | 53 (37%) | 67 (47%) | 22 (16%) | 2004-2016 |
| **6** | 44 (47%) | 38 (41%) | 11 (12%) | 2001-2015 |
| **7** | 147 (42%) | 180 (51%) | 23 (7%) | 2000-2015 |
| **8** | 144 (48%) | 106 (35%) | 52 (17%) | 1997-2016 |

[Supplemental Table 1] Time-frame per hospital.
