# Supplementary material for: Arteriovenous Fistula Maturation Failure in a Large Cohort of Hemodialysis Patients in the Netherlands
Source: World J Surg. 2017 Nov 29;42(6):1895–903. doi: 10.1007/s00268-017-4382-z (PMC5934452; doi:10.1007/s00268-017-4382-z)
Supplement: Supplementary file 3 — Supplementary material 3 (DOCX 16 kb) [file 268_2017_4382_MOESM3_ESM.docx]

|  | **RCAVF (n=663)** | **Upper arm AVF (n=699)** | **AVG (n=243)** |
| --- | --- | --- | --- |
| **Known outcome** | 617 (93.1%) | 650 (93.0%) | 229 (94.2%) |
| Successful maturation | 468 (70.6%) | 581 (83.1%) | 216 (88.9%) |
| AVF nonmaturation/AVG functional failure | 149 (22.5%) | 69 (9.9%) | 13 (5.3%) |
| **Unknown outcome** | 46 (6.9%) | 49 (7.0%) | 14 (5.8%) |
| Ligation for HAIDI | 4 (0.6%) | 3 (0.4%) | 0 (0%) |
| Other reasons related to VA | 1 (0.2%) | 2 (0.3%) | 0 (0%) |
| Recovery of renal function | 23 (3.5%) | 20 (2.9%) | 5 (2.1%) |
| Transplantation | 4 (0.6%) | 1 (0.1%) | 0 (0%) |
| Death | 9 (1.4%) | 11 (1.6%) | 1 (0.4%) |
| Other reasons not related to VA | 5 (0.8%) | 12 (1.7%) | 8 (3.3%) |

[Supplemental Table 2] Outcomes of maturation or functional failure for the entire cohort. In case of an unknown outcome, neither imaging nor cannulation was performed prior to abandonment of the VA for the given reason.
