# Supplementary material for: Arteriovenous Fistula Maturation Failure in a Large Cohort of Hemodialysis Patients in the Netherlands
Source: World J Surg. 2017 Nov 29;42(6):1895–903. doi: 10.1007/s00268-017-4382-z (PMC5934452; doi:10.1007/s00268-017-4382-z)
Supplement: Supplementary file 4 — Supplementary material 4 (DOCX 16 kb) [file 268_2017_4382_MOESM4_ESM.docx]

|  | **RCAVF (n=617)** | | **Upper arm AVF (n=650)** | | **AVG (n=229)** | |
| --- | --- | --- | --- | --- | --- | --- |
| **VA outcome** | 3 months | 6 months | 3 months | 6 months | 3 months | 6 months |
| **Failed** | 165 (26.7%) | 179 (29.0%) | 84 (12.9%) | 97 (14.5%) | 30 (13.1%) | 41 (17.9%) |
| Primary failure | 149 (24.1%) | 149 (24.1%) | 69 (10.6%) | 69 (10.6%) | 13 (5.7%) | 13 (5.7%) |
| Loss of VA within time frame | 16 (2.6%) | 30 (4.9%) | 15 (2.3%) | 28 (4.3%) | 17 (7.4%) | 28 (12.2%) |
| **Successful use during time frame** | 411 (66.6%) | 367 (59.5%) | 506 (77.8%) | 452 (69.5%) | 178 (77.7%) | 157 (68.6%) |
| **Indeterminate** | 41 (6.6%) | 71 (11.5%) | 60 (9.2%) | 101 (15.5%) | 21 (9.2%) | 31 (13.5%) |

[Supplemental Table 3] AVF maturation and AVG functional outcomes defined as achieving 3- and 6-months of functional patency. Indeterminate denotes VAs used but lost to follow-up or abandoned for death, recovery of renal function or transplantation before 3 and 6 months, respectively.
