# Supplementary material for: Arteriovenous Fistula Maturation Failure in a Large Cohort of Hemodialysis Patients in the Netherlands
Source: World J Surg. 2017 Nov 29;42(6):1895–903. doi: 10.1007/s00268-017-4382-z (PMC5934452; doi:10.1007/s00268-017-4382-z)
Supplement: Supplementary file 5 — Supplementary material 5 (DOCX 16 kb) [file 268_2017_4382_MOESM5_ESM.docx]

|  | **First procedure (n=264)** | **Second procedure (n=66)** | **Third procedure (n=16)** |
| --- | --- | --- | --- |
| **Type** |  |  |  |
| Balloon angioplasty | 136 (51.5%) | 45 (68.2%) | 12 (75%) |
| Stenting | 0 (0%) | 3 (4.5%) | 0 (0%) |
| Thrombectomy | 26 (9.8%) | 6 (9.1%) | 0 (0%) |
| Other endovascular | 1 (0.4%) | 0 (0%) | 2 (12.5%) |
| Surgical, revision | 85 (32.2%) | 10 (15.2%) | 2 (12.5%) |
| Surgical, other | 10 (3.8%) | 0 (0%) | 0 (0%) |
| Unknown | 6 (2.3%) | 2 (3.0%) | 0 (0%) |
| **Location** |  |  |  |
| Artery | 19 (7.2%) | 2 (3.0%) | 0 (0%) |
| Anastomosis | 152 (57.6%) | 38 (57.6%) | 11 (68.8%) |
| Graft | 25 (9.5%) | 7 (10.6%) | 1 (6.3%) |
| Vein | 56 (21.2%) | 12 (18.2%) | 2 (12.5%) |
| Central vein | 3 (1.1%) | 5 (7.6%) | 2 (12.5%) |
| Unknown | 9 (3.4%) | 2 (3.0%) | 0 (0%) |

[Supplemental Table 4] Types and locations of procedures to promote maturation or functional success.
