# Supplementary material for: Arteriovenous Fistula Maturation Failure in a Large Cohort of Hemodialysis Patients in the Netherlands
Source: World J Surg. 2017 Nov 29;42(6):1895–903. doi: 10.1007/s00268-017-4382-z (PMC5934452; doi:10.1007/s00268-017-4382-z)
Supplement: Supplementary file 6 — Supplementary material 6 (DOCX 16 kb) [file 268_2017_4382_MOESM6_ESM.docx]

| **Hospital** | **RCAVF** | **Upper arm AVF** | **AVG** |
| --- | --- | --- | --- |
| **1** | 50% (12/24) | 61% (19/31) | 75% (3/4) |
| **2** | 60% (25/42) | 76% (22/29) | 97% (59/61) |
| **3** | 62% (13/21) | 80% (72/90) | 90% (9/10) |
| **4** | 69% (22/32) | 78% (28/36) | 89% (8/9) |
| **5** | 48% (10/21) | 33% (9/27) | 73% (8/11) |
| **6** | 70% (16/23) | 70% (14/20) | 100% (9/9) |
| **7** | 59% (38/64) | 77% (78/102) | 95% (18/19) |
| **8** | 67% (40/60) | 74% (51/69) | 88% (28/32) |

[Supplemental Table 5] 3-month cannulation rates in prevalent HD patients, per hospital.
