# Supplementary material for: Arteriovenous Fistula Maturation Failure in a Large Cohort of Hemodialysis Patients in the Netherlands
Source: World J Surg. 2017 Nov 29;42(6):1895–903. doi: 10.1007/s00268-017-4382-z (PMC5934452; doi:10.1007/s00268-017-4382-z)
Supplement: Supplementary file 7 — Supplementary material 7 (DOCX 17 kb) [file 268_2017_4382_MOESM7_ESM.docx]

|  | **RCAVF** | | **Upper arm AVF** | | **AVG** | |
| --- | --- | --- | --- | --- | --- | --- |
| **Time period** | Nonmaturation | Maturation | Nonmaturation | Maturation | Functional failure | Functional success |
| 1997-1999 | 1 (33%) | 2 (67%) | 0 | 0 | 0 | 2 (100%) |
| 2000-2004 | 18 (19%) | 75 (81%) | 6 (12%) | 45 (88%) | 2 (10%) | 18 (90%) |
| 2005-2009 | 44 (24%) | 141 (76%) | 19 (11%) | 155 (89%) | 4 (6%) | 68 (94%) |
| 2010-2014 | 76 (25%) | 232 (85%) | 38 (10%) | 335 (90%) | 4 (3%) | 114 (97%) |
| 2015-2016 | 10 (36%) | 18 (64%) | 6 (12%) | 46 (88%) | 3 (18%) | 14 (82%) |

[Supplemental Table 6] Trends over time in maturation and primary failure of AVFs and AVGs
